# Chemoprevention of intestinal tumorigenesis by nabumetone: induction of apoptosis and Bcl-2 downregulation

**DOI:** 10.1054/bjoc.2001.1807

**Published:** 2001-05

**Authors:** H K Roy, W J Karoski, A Ratashak, T C Smyrk

**Affiliations:** Department of Internal Medicine, University of Nebraska Medical Center/Eppley Cancer Institute, 982000 Nebraska Medical Center, Omaha NE, 68198-2000, USA; Department of Pathology, Mayo Clinic, 200 N First Street, Rochester MN, USA

**Keywords:** colon cancer, nonsteroidal anti-inflammatory drugs, apoptosis

## Abstract

Treatment of MIN mice with the nonsteroidal anti-inflammatory drug, nabumetone, resulted in a dose-dependent suppression of intestinal tumorigenesis. In both the uninvolved MIN mouse colonic epithelium and HT-29 colon cancer cells, nabumetone downregulated the anti-apoptotic protein, Bcl-2, with concomitant induction of apoptosis, suggesting a potential mechanism for colon cancer chemoprevention. © 2001 Cancer Research Campaign www.bjcancer.com


*British Journal of Cancer* (2001) **84**(10), 1412–1416
© 2001 Cancer Research Campaign
doi: 10.1054/ bjoc.2001.1807, available online at http://www.idealibrary.com on IDEAL

### Chemoprevention of intestinal tumorigenesis by nabumetone: induction of apoptosis and Bcl-2 downregulation

**HK Roy[1], WJ Karoski[1], A Ratashak[1] and TC Smyrk[2]**

[1]Department of Internal Medicine, University of Nebraska Medical Center/Eppley Cancer Institute, 982000 Nebraska Medical Center, Omaha NE, 68198-2000, USA; [2]Department of Pathology, Mayo Clinic, 200 N First Street, Rochester MN, USA

**Summary** Treatment of MIN mice with the nonsteroidal anti-inflammatory drug, nabumetone, resulted in a dose-dependent suppression of intestinal tumorigenesis. In both the uninvolved MIN mouse colonic epithelium and HT-29 colon cancer cells, nabumetone downregulated the anti-apoptotic protein, Bcl-2, with concomitant induction of apoptosis, suggesting a potential mechanism for colon cancer chemoprevention.
© 2001 Cancer Research Campaign http://www.bjcancer.com

**Keywords**: colon cancer; nonsteroidal anti-inflammatory drugs; apoptosis

Epidemiological and experimental studies indicate that nonsteroidal anti-inflammatory drugs (NSAIDS) confer up to a 50% risk-reduction against colorectal cancer (Janne and Mayer, 2000). Nevertheless, there has been a reluctance to utilize this strategy in clinical practice, largely due to concerns of potential gastrointestinal toxicity. To decrease gastrointestinal toxicity, NSAIDS have been developed with selectivity for cyclooxygenase (COX) 2, the isoform of the prostaglandin-producing enzyme not expressed in normal gastrointestinal mucosa (Warner et al, 1999), but upregulated early during colon carcinogenesis. Nabumetone has a seven-fold higher selectivity for COX 2 than COX 1 (DeWitt et al, 1993) and clinical studies indicate that it is 10–36 times safer than conventional NSAIDS (Huang et al, 1999). However, data regarding nabumetone's ability to protect against colon cancer is quite limited.

In the current study, we evaluated the chemopreventive efficacy of nabumetone using the MIN mouse, the murine equivalent of familial adenomatous polyposis (FAP), in which germline mutations in the adenomatous polyposis coli (APC) gene lead to multiple intestinal adenomas. APC truncations initiate most colorectal cancers, underscoring the biological relevance of this model. To investigate potential mechanisms of chemoprevention, we evaluated nabumetone's ability to induce apoptosis and downregulate Bcl-2 both in the MIN mouse and the human colon cancer cell line HT-29.

### METHODS

### MIN mice

Approval was obtained from the Institutional Animal Care Utilization Committee of the University of Nebraska Medical Center. Twenty-four male MIN mice, 5–6 weeks old (Jackson Labs, Bar Harbor, ME) were randomized to either a standard AIN 93a diet alone (Harlan Teklad Labs, Madison, WI) or with nabumetone supplementation at 300 ppm, 600 ppm, or 900 ppm (kindly provided by SmithKline Beecham, Collegeville, PA). After 10 weeks on diet, the animals were sacrificed and intestinal tumours were scored under magnification by an observer blinded to treatment group. Intestinal scrapings and sections were taken at sacrifice.

*Received 30 August 2000*
*Revised 23 February 2001*
*Accepted 26 February 2001*

*Correspondence to: HK Roy*

### Cell culture

HT-29 cells (American Type Cell Culture, Rockville, MD) were grown in Dulbeccos Modified Essential Media Cells with 10% fetal calf serum. Apoptosis was quantitated via flow cytometric determination of the subdiploid fraction of propridium iodine treated cells. To confirm apoptosis, we utilized the M30 CytoDEATH antibody (Boeringer Manheim, Indianapolis, IN), which targets a neo-epitope produced from cleavage of cytokeratin 18 by caspase 3 (Carr, 2000).

### Western blot analysis

Immunoblot analysis was performed on intestinal homogenates and HT-29 lysates as previously described (Roy et al, 1995). Membranes were probed with a polyclonal antibody to Bcl-2 (Oncogene Science, Cambridge, MA) or β-actin (Santa Cruz Biotechnology, Santa Cruz, CA), the latter serving as a gel loading control along with examination of India Ink stained membranes.

### Tissue section analysis

Apoptotic cells were identified using a modified terminal deoxynucleotidyl-transferased UTP nick end labelling (TUNEL) assay (Frag El-Klenow kit, Oncogene Science, Cambridge, MA). Immunohistochemistry was performed with a polyclonal antibody to Bcl-2 (Santa Cruz Biotechnology, Santa Cruz, CA) or the

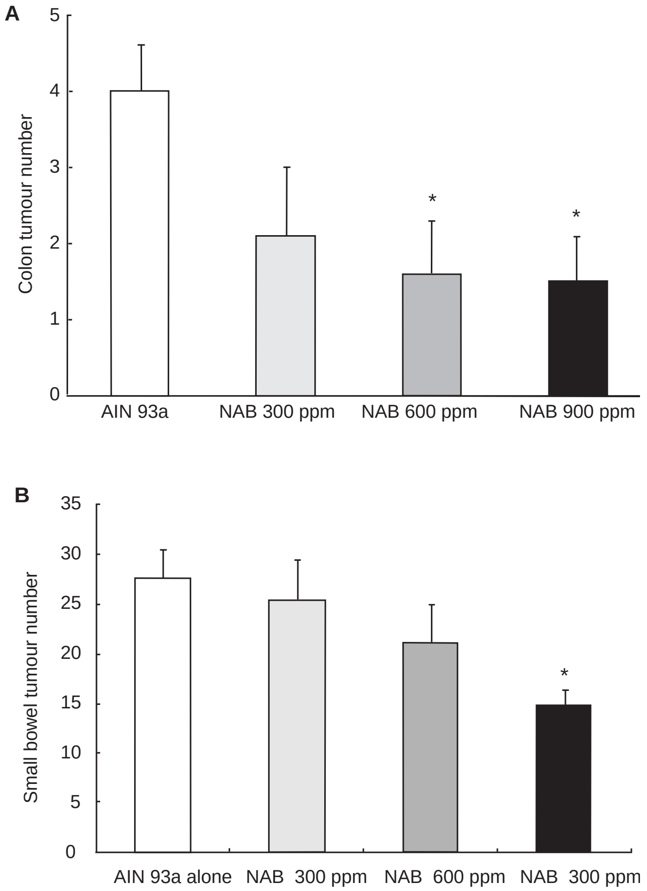

**Figure 1** Effect of nabumetone supplementation on (**A**) colonic and (**B**) small bowel tumour number. Nabumetone caused a dose-dependent reduction in tumors (* *P* < 0.05 versus control diet)

monoclonal M30 antibody. Slides were developed with the Vectastain A, B, C kit (Vector Laboratories, Burlingame, CA).

### RESULTS

### Nabumetone inhibited MIN mouse tumorigenesis

There were 31.6 ± 3.4 intestinal tumours in the MIN mice fed the control, AIN 93a diet alone, 87% of which were in the small intestine. All tumours sampled for histology were adenomas. Nabumetone caused a dose-dependent reduction in small intestinal and colonic tumours (Figure 1).

### Nabumetone induced apoptosis in MIN mice and HT-29 cells

Based on our tumour data, we focused on the control versus 900 ppm nabumetone group for all subsequent analysis. Nabumetone approximately doubled the apoptosis rate in the uninvolved colonic epithelium (Figure 2A). This TUNEL data was confirmed in representative tissue sections by immunohistochemical detection of the M30 cytoDEATH epitope (data not shown), which is specific for an earlier stage in apoptosis in the colon (Carr, 2000). In HT-29 cells, nabumetone caused a dose-dependent induction of apoptosis ($r^2$ = 0.995) (Figure 2B) by subdiploid fraction analysis and was also duplicated with the M30 antibody (Figure 2C).

### Nabumetone downregulates Bcl-2 expression

Nabumetone decreased expression of Bcl-2 in the uninvolved and adenomatous MIN mouse mucosa (Figure 3). Immunohistochemical analysis suggested that this was predominantly epithelial (Figure 3C). In HT-29 cells, nabumetone's Bcl-2 downregulation paralleled the induction of apoptosis (Figure 4).

### DISCUSSION

Nabumetone supplementation was well-tolerated with no evidence of toxicity or alteration in body weight. This is consistent with clinical observations (Huang et al, 1999) and results from animal studies which indicate that nabumetone is much less toxic than conventional NSAIDS.

Nabumetone caused a dramatic, dose-dependent reduction in MIN mouse tumorigenesis which was comparable to reports with other NSAIDS in this model. For instance, 200 ppm sulindac resulted in a 17% tumour reduction (Torrance et al, 2000) whereas the maximal tolerated dose (320 ppm) suppressed 70% of tumours (Beazer-Barclay et al, 1996). Furthermore, celecoxib, a selective COX 2 inhibitor, reduced tumour multiplicity by 50–71% (Jacoby et al, 2000). Therefore, our demonstration that 900 ppm nabumetone inhibited small bowel and colonic tumours by 50% and 65%, respectively, compares favourably with other NSAIDS. Moreover, given the lack of toxicity, it is conceivable that higher doses could be used with potentially greater efficacy.

The data presented herein is the first to demonstrate nabumetone's efficacy in the protection against intestinal tumorigenesis. While more selective COX 2 inhibitors are available, the modest

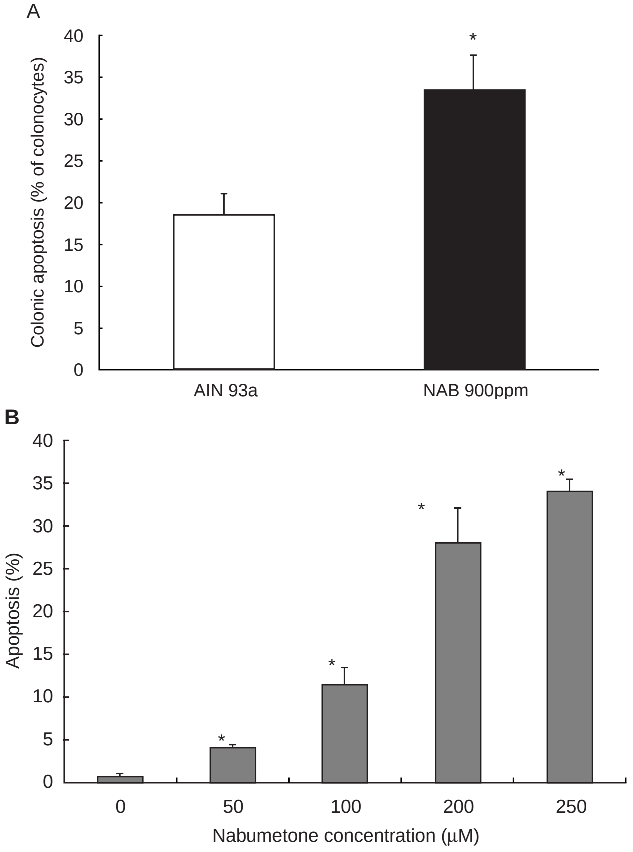

**Figure 2** (Continued)

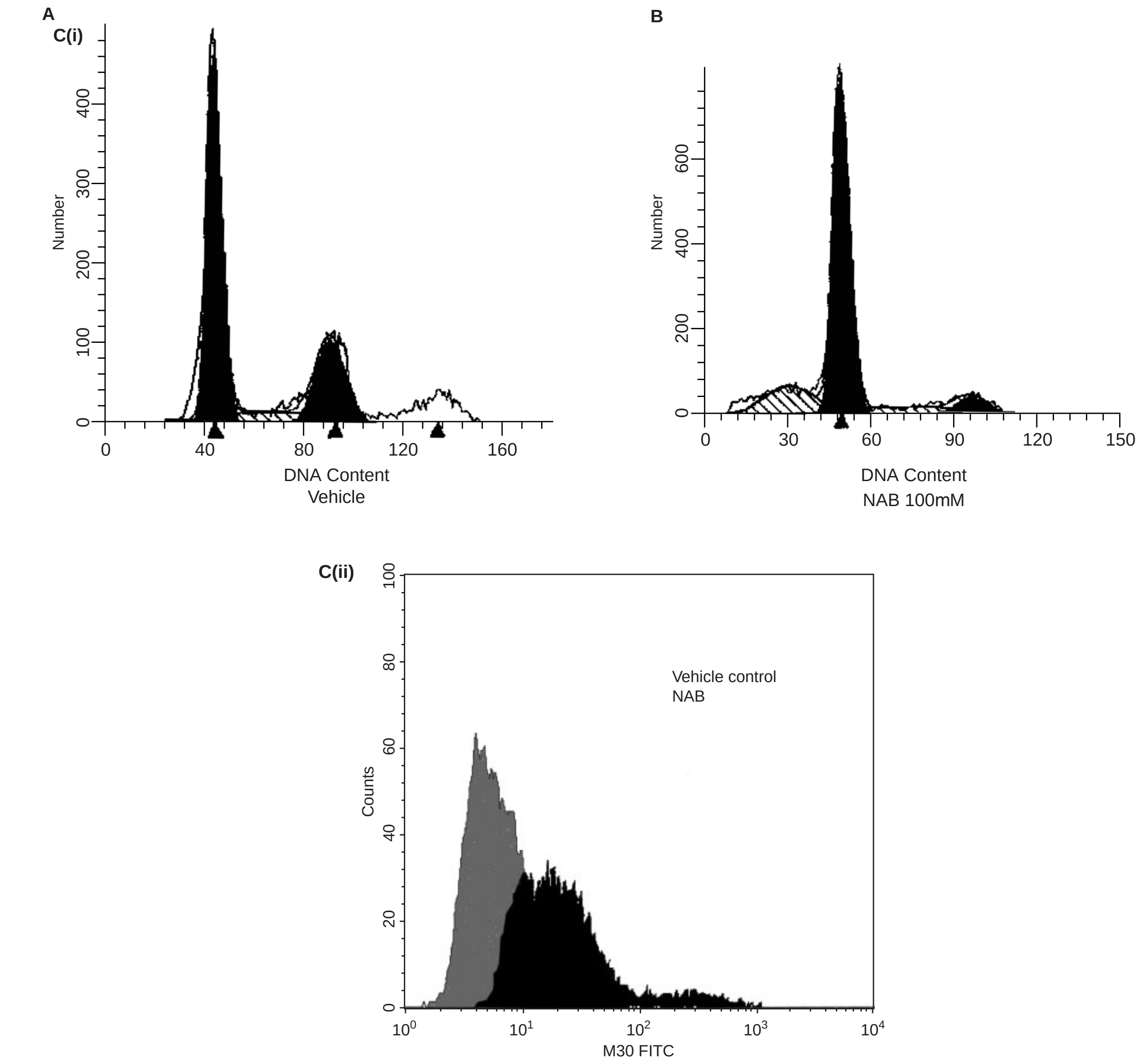

**Figure 2** Nabumetone induced apoptosis in both the MIN mice and the human colon cancer cell line HT-29. (**A**) Apoptosis in the uninvolved colonic mucosa was assessed by a modified TUNEL assay in MIN mice on the AIN 93a diet alone compared to those supplemented with 900 ppm nabumetone. Data is expressed as % apoptotic cells (mean ± S.E.) (* $P < 0.01$). (**B**) Nabumetone induction of apoptosis in HT-29 cells was dose-dependent. Subdiploid fraction was assessed at 7 days of treatment with nabumetone and expressed as % apoptotic cells (mean ± S.E.) (*$P < 0.05$ versus control). (**C**) Representative flow cytometric apoptosis analysis utilizing; (i) Subdiploid fraction; (ii) Fraction of cells displaying the M30 epitope. Grey represents vehicle control while black are nabumetone-treated cells inhibition of COX 1 by nabumetone may have advantages in protection against both colon cancer (Chaluda et al, 2000) and cardiovascular diseases. With regards to the latter, it has been demonstrated that highly selective COX 2 inhibition may potentially increase cardiac risk (Bombardier et al, 2000) presumably by increasing platelet aggregation via inhibition of anti-aggregatory prostacyclin (derived from endothelial COX 2) while not targeting the prothrombotic platelet COX 1 produced thromboxane (McAdams et al, 1999). Therefore, nabumetone, with its safety and anti-platelet activity would have advantages from a public health perspective over conventional as well as highly COX 2 selective NSAIDS.

To investigate the potential mechanism(s) involved in chemoprevention, we evaluated apoptosis in the uninvolved mucosa. The inhibition of apoptosis is crucial in allowing the otherwise short-lived colonocytes to accumulate the requisite mutations for neoplastic transformation. The induction of apoptosis by nabumetone was comparable to reports with other NSAIDS in this model (Boolbol et al, 1996). Furthermore, our MIN mouse data is reinforced by the demonstration that nabumetone treatment of HT-29 cells resulted in a dose-dependent induction of apoptosis.

The regulation of intestinal apoptosis is complex and multifactorial. However, recent data underscores the paramount role of the Bcl-2 family. The relative abundance of anti-and pro-apoptotic members govern the cellular propensity towards apoptosis (Tsujimoto and Shimizu, 2000). Nabumetone's downregulation of the anti-apoptotic Bcl-2 was more marked in uninvolved mucosa then in adenomas, a finding consistent with its potential role in the early stages of carcinogenesis (Baretton et al, 1996). Our immunohistochemical analysis

 *© 2001 Cancer Research Campaign*

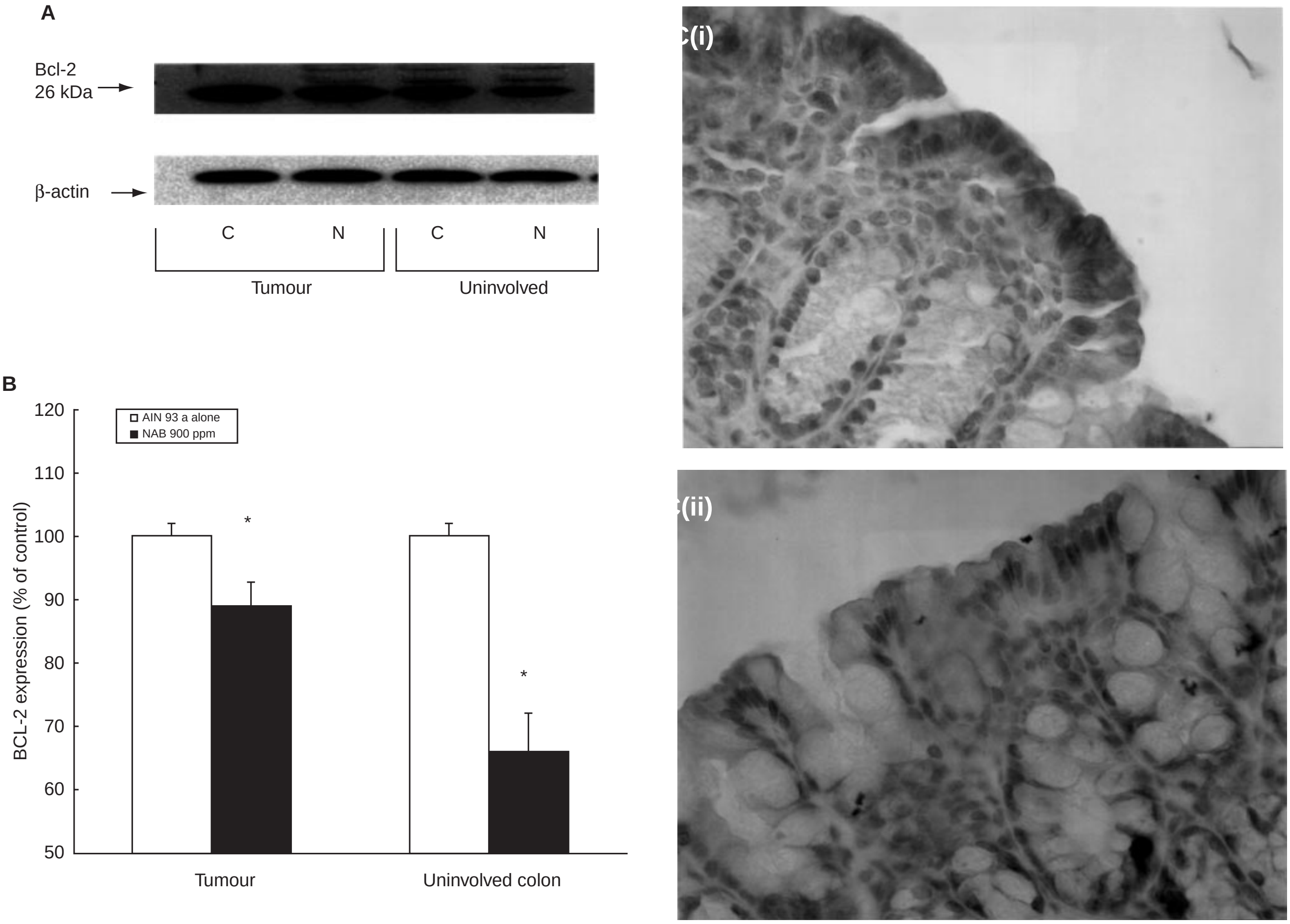

**Figure 3** The effect of nabumetone 900 ppm supplementation on Bcl-2 was assessed in uninvolved MIN mouse colonic mucosa as well as tumours. (**A**) Representative Western blot of Bcl-2. (**B**) Densitometric analysis of Bcl-2 expression, normalized to AIN 93a diet alone. Data expressed as mean ± S.E. (* *P* < 0.05 versus control). (**C**) Representative immunohistochemistry: (i) Uninvolved colon mucosa from animals on a control diet; (ii) Uninvolved colon mucosa from animals treated with NAB 900 ppm

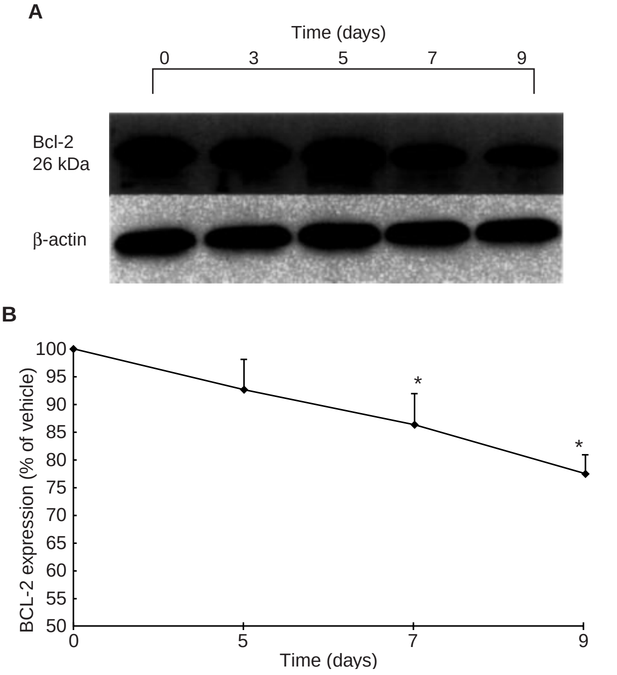

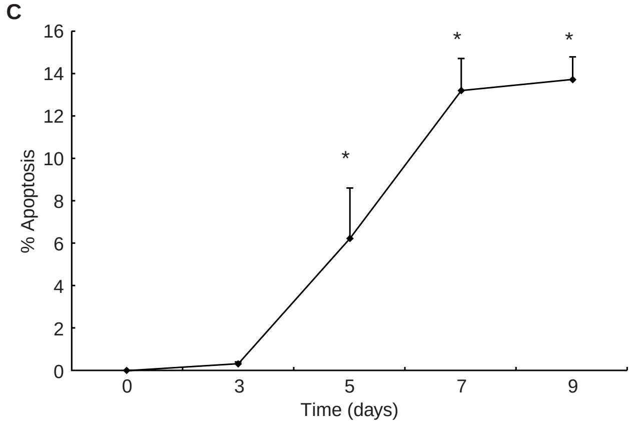

**Figure 4** Effect of nabumetone on Bcl-2 expression and apoptosis in HT-29 cells. Cells were treated with 100 μM NAB for the indicated time and expression assessed by immunoblot analysis. (**A**) Representative Western blot of Bcl-2. (**B**) Densitometric analysis of Bcl-2 expression normalized to the appropriate vehicle control. (**C**) Corresponding time-dependent apoptosis rate (by subdiploid fraction) in the HT-29 cells treated with 100 μM NAB. These were adjusted for the low level of apoptosis (< 2%) in the vehicle treated cells. Data expressed as mean ± S.E. (* *P* < 0.05 versus control)

*© 2001 Cancer Research Campaign*

qualitatively supports the Western blot analysis (Figure 2). Furthermore, we demonstrate that nabumetone downregulation of Bcl-2 in HT-29 cells mirrored induction of apoptosis. Our data is in agreement with the demonstration that sulindac reduced Bcl-2 expression in both the uninvolved rectal mucosa of patients with FAP (Winde et al, 1997) and in MIN mouse adenomas (McEntee et al, 1999). The mechanism by which NSAIDS downregulate Bcl-2 is unclear, with one report implicating prostaglandin E2, (Sheng et al, 1998) although several lines of evidence suggesting that prostaglandin inhibition may not be essential for either NSAID-induced apoptosis or chemoprevention (Williams et al, 1997). While Bcl-2 is clearly of importance in governing colonocyte apoptosis (Hague et al, 1998), recent studies have also implicated pro-apoptotic members of the family. For instance, Bak expression correlates with apoptosis rates in the normal human colon (Liu et al, 1999). However, in HCT-116, a microsatellite unstable colon cancer cell line, NSAIDS altered the apoptotic threshold by downregulating the anti-apoptotic Bcl-$X_L$ without changing expression of the pro-apoptotic Bax (Zhang et al, 2000). This is clearly analogous to our data with nabumetone in a microsatellite stable cell line.

In summary, we demonstrate that nabumetone is a safe and effective as a colon cancer chemopreventive agent, potentially through induction of apoptosis. While the mechanisms are still not completely elucidated, our data suggests that Bcl-2 downregulation may be important in this process.

### REFERENCES

Baretton GB, Diebold J, Cristoforis G, Vogt M, Muller C, Doffer K, Schneiderbanger K, Schmidt M and Lohrs U (1996) Apoptosis and immunohistochemical bcl-2 expression in colorectal adenomas and carcinomas: Aspects in carcinogenesis and prognostic significance. *Cancer* **77**: 255–264

Barnes CJ, Hardman EE and Lee M (1998) Chemoprevention of spontaneous intestinal adenomas in the adenomatous polyposis coli Min mouse model with aspirin. *Gastro* **114**: 873–877

Beazer-Barclay Y, Levy DB, Moser AR, Dove WF, Hamilton SR, Vogelstein B and Kinzer KW (1996) Sulindac suppresses tumorigenesis in the Min mouse. *Carcinogenesis* **17**: 1757–1760

Bombardier C, Laine L, Reicin A, Shapiro D, Burgos-Vargas R, Davis B, Day R, Ferraz MB, Hawkey CJ, Hochberg MC, Kvein TK, Schnitzer TJ and Weaver A (2000) Comparison of upper gastrointestinal toxicity of rofecoxib and naproxen in patients with rheumatoid arthritis. *N Engl J Med* **343**: 1520–1528

Boolbol SK, Danneberg AJ, Chadburn A, Martucci C, Guo XJ, Ramonetti JT, Abreu-Goris M, Newmark HL, Lipkin ML, DeCrosse JJ and Bertagnolli MM (1996) Cycloxygenase-2 over-expression and tumor formation are blocked by sulindac in a murine model of familial adenomatous polyposis. *Cancer Res* **56**: 2556–2560

Carr NJ (2000) M30 Expression demonstrates apoptotic cells, correlates with in situ end-labeling and is associated with Ki-67 expression in large intestinal neoplasms. *Arch Pathol Lab Med* **124**: 1768–1772

Chulada PC, Thompson MB, Mahler JF, Doyle CM, Gaul BW, Lee C, Tiano HF, Morham SG, Smithies O and Langenbach R (2000) Genetic Disruption of *ptgs-1*, as well as *ptgs-2* Reduces Intestional Tumorigenesis in the Min mice. *Cancer Res* **60**: 4705–4708

Chung DC (2000) The genetic basis of colorectal cancer: insights into critical pathways of tumorigenesis. *Gastroenterol* **119**: 854–865

Hague A, Bracey TS, Hicks DJ, Reed JC and Paraskeva C (1998) Decreased levels of p26-Bcl-2, but not p30 phosphorylated Bcl-2, precede TGF$\beta_1$-induced apoptosis in colorectal adenoma cells. *Carcinogenesis* **19**(9): 1691–1695

Huang JQ, Sridhar S and Hunt RH (1999) Gastrointestinal safety profile of nabumetone: a meta-analysis. *Am J Med* **107**: 55S–61S

Jacoby RG, Seifert K, Cole CE, Kelloff G and Lubet RA (2000) The cyclooxygenase-2 inhibitor celecoxib is a potent preventive and therapeutic agent in the min mouse models of adenomatous polyposis *Cancer Res* **260**: 5040–5044

Janne PA and Mayer RJ (2000) Chemoprevention of colorectal cancer. *N Engl J Med* **342**: 1960–1968

Liu LU, Holt PR, Krivosheyev V and Moss SF (1999) Human right and left colon differ in epithelial apoptosis and in expression of Bak, a pro-apoptotic Bcl-2 homologue. *Gut* **45**: 45–50

McAdams BF, Catella-Lawson F, Mardini IA, Kapoor S, Lawson JA and FitzGerald GA (1999) Systemic biosynthesis of prostacyclin by cyclooxygenase (COX)-2: the human pharmacology of selective inhibitor of COX-2. *Proc Natl Acad Sci USA* **96**: 272–277

McEntee MF, Chiu CH and Whelan J (1999) Relationship of beta-catenin and Bcl-2 expression to sulindac-induced regression of intestinal tumors in Min mice. *Carcinogenesis* **20**: 635–640

Roy HK, Bissonnette M, Frawley BP Jr, Wali RK, Niedziela SM, Earnest D and Brasitus TA (1995) Selective preservation of protein kinase C-zeta in the chemoprevention of azoxymethane-inducedcolonic tumors by piroxicam. *FEBS Lett* June 12; **366**(2–3): 143–145

Sheng H, Shao J, Morrow JD, Beauchamp RD and DuBois RN (1998) Modulation of apoptosis and Bcl-2 expression by prostaglandin $E_2$ in human colon cancer cells. *Cancer Res* **58**: 362–366

Torrance CJ, Jackson PE, Montgomery E, Kinzler KW, Vogelstein B, Wissner A, Nunes M, Frost P and Discafani CM (2000) Combinatorial chemoprevention of intestinal neoplasia. *Nature Medicine* **6**: 1024 – 1028

Tsujimoto Y and Shimizu S (2000) Bcl-2 family: life-or-death switch. *FEBS Lett* Jan 21; **466**(1): 6–10

Warner TD, Giuliano F, Vojnovic I, Bukasa A, Mitchell JA and Vane JR (1999) Nonsteroid drug selectivities for cyclo-oxygenase—1 rather than cyclo-oxygenase-2 are associated with human gastrointestinal toxicity: a full in vitro analysis. *Proc Natl Acad Sci USA* **96**: 7563–7568

Williams CS, Smalley W and DuBois RN (1997) Aspirin use and potential mechanisms for colorectal cancer prevention. *J Clin Invest* **100**: 1325–1329

Winde G, Schmid KW, Brandt B, Muller O and Osswald H (1997) Clinical and Genomic Influence of sulindac on rectal mucosa in familial adenomatous polyposis. *Dis Colon Rectum* **40**: 1156–1168

Zhang L, Yu J Park BH, Kinzler KW and Vogelstein B (2000) Role of BAX in the apoptotic response to anticancer agents. *Science* **290**: 989–992

*© 2001 Cancer Research Campaign*
